# Nasal spindle cell sarcoma: A case report

**DOI:** 10.1016/j.ijscr.2020.05.092

**Published:** 2020-06-11

**Authors:** Mohammad Almahdi, Salwa ALRashed ALHumaid, Maryam Alsafi, Fahad AlObaid, Alaa Alsalim

**Affiliations:** a Division of Otolaryngology–Head and Neck Surgery, Department of Surgery, King Abdulaziz Medical City and King Abdullah Specialist Children’s Hospital, National Guard Health Affairs, Riyadh, Saudi Arabia; b Department of Pathology and Laboratory Medicine, King Abdulaziz Medical City and King Abdullah Specialist Children’s Hospital National Guard Health Affairs, Riyadh, Saudi Arabia

**Keywords:** Spindle cell sarcoma, Monophonic synovial sarcoma, Nasal cavity, Head and neck, Functional endoscopic sinus surgery, Cranial tumor resection

## Abstract

**Background:**

Synovial sarcoma is a mesenchymal high grade tumor. It is found in soft tissue mostly lower extremities. It is rarely found in the head and neck area with paraspinal neck accounting for the most common location. In rare occasions, it can be found in nasal cavity.

**Case presentation:**

A 46-year-old female patient presented with recurrent epistaxis for 7 months. A 0-degree rigid scope examination revealed a left nasal cavity mass. A non-contrasted CT scan of the paranasal sinus showed heterogeneous and hypodense lesion in the left nasal cavity. Functional endoscopic sinus surgery was performed and multiple nasal-mass biopsies were taken which showed high grade spindle cell sarcoma favoring synovial type. Further workup and management were done from MRI, cranial tumor resection with skull base reconstruction to adjuvant radiotherapy. Patient has regular follow up with involved specialties and thus far is doing well.

**Conclusion:**

Spindle cell synovial sarcoma is a rare lesion found in soft tissue. It is unusual to be found in nasal cavity. Thus crucial attention to the clinical, radiographical and histopathological manifestation should be taken for more accurate diagnosis and further appropriate management.

## Highlights

• Synovial sarcoma is a mesenchymal high grade tumor.
• It is rarely found in the head and neck area, nevertheless in nasal cavity.
• Nasal manifestations may range from nasal obstruction, discharge, foul smell to epistaxis.
• Crucial attention to the manifestation should be taken for accurate diagnosis and appropriate management.

## 1 Introduction

Synovial sarcoma is a mesenchymal spindle cell tumor found in soft tissue. It is a rare tumor that is unlike its name not related to synovial membrane. It is mostly found in lower extremities. It accounts 5–10% of all adult sarcoma. Around 5–15% of synovial sarcoma can be found in head and neck areas.

It is considered as a high grade malignant tumor. The paraspinal neck accounts for the most common location of this neoplasm in the head and neck region.

However, it could also be found in nasopharynx, oropharynx and thyroid gland in some cases with rare occasions found in nasal cavity and maxilla. No gender predisposition was found in synovial sarcoma. However, it seems to affect patients mostly in their third decade. Presenting symptoms are mostly determined by location of tumor. Management depends mostly on surgical resection with consideration of radiotherapy. Prognosis of head and neck synovial sarcoma has similar rates of all sarcomas with a 5 year-survival rate reaching up to 50% [[1], [2], [3]].

This project has been reported according to the SCARE criteria [4].

## 2 Case presentation

A 46-year-old female, known case of rheumatoid arthritis on Methotrexate, hypothyroidism on Levothyroxine and unremarkable surgical history referred to otolaryngology clinic as a case of recurrent epistaxis from the left nostril for the last 7 months, required nasal packing at one point. Her chief complaint was associated with hyposmia, nasal discharge, nasal obstruction, left ear fullness, mouth breathing and dryness. She had intentional weight loss, insignificant for the past 3 months, decrease in appetite with on and off night sweat.

She had unremarkable family history except of a sister with breast cancer. She had no known allergies. Upon examination, 0-degree rigid nasal scope was used and showed an easily bleeding swelling. It was blocking the left nostril with difficulty in determining its origin whether it was from the septum or lateral nasal wall. Scope through the right side revealed patent nasal cavity and clear nasopharynx.

**Fig. 1:**
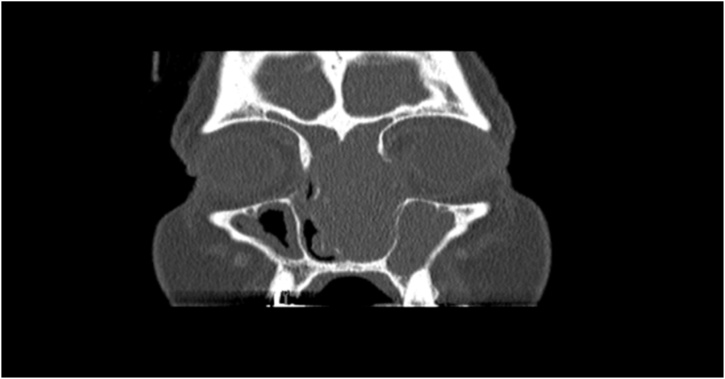
Preoperative CT coronal Cut showed left nasal mass with bony invasion.

Urgent sinus computed tomography (CT) revealed large left nasal cavity heterogeneous and hypodense mass with faint central enhancement. It was seen extending to the left maxillary sinus, with opacification of anterior ethmoid air cells and left frontal sinus and widening of the left osteo-meatal unit. It showed as well severe thinning of the left maxillary sinus wall, nasal septum and inferior left orbital wall with possible dehiscence of the left lamina papyracea, bilateral cribriform plate and medial part of the fovea ethmoidalis with clear nasopharynx (Fig. 1).

Patient underwent examination of nasal cavity under general anaesthesia and debulking of the left nasal mass. Left nasal cavity was completely obstructed with a nasal mass, its origin could not be identified precisely but it was seen below and separable from the middle turbinate. It seemed attached to the inferior turbinate and to the nasal septum. Multiple biopsies were taken for histopathology and debulking was done using a debrider and forceps until reaching the nasopharynx which was clear from any obstruction. After that, left middle sac was opened through the natural ostium which revealed some secretions.

**Fig. 2:**
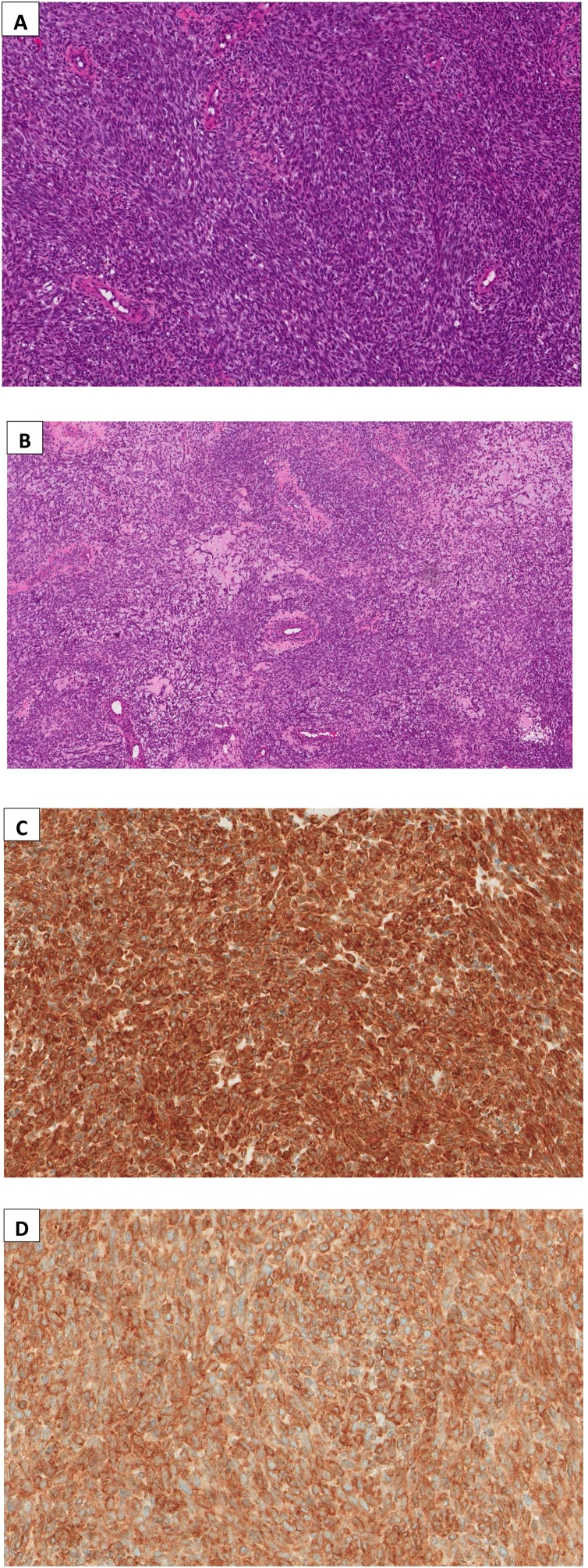
(A) Monophasic synovial sarcoma with uniform spindle cells arranged in solid sheets. (B) Perivascular hyalinization and myxoid change. Immunohistochemistry performed on the biopsy shows diffuse staining for (C) EMA and (D) Bcl-2.

On histological examination, the biopsy showed monotonous population of plump to spindle cells with indistinct cytoplasm and oval dark stained nuclei, arranged in solid sheets and short fascicles. These are alternating with less cellular areas showing myxoid change and perivascular hyalinization (Fig. 2 A&B). The tumor shows few mitotic figures. The tumor cells are positive for EMA (Fig. 2. C) and Bcl-2 (Fig. 2. D) with focal immunoreactivity to SMA, while being negative for S100, desmin, myogenin, myo D1, CD99, SOX-10 and CD34. The morphological features and the immunoprofile of the tumor are highly diagnostic of monophasic synovial sarcoma.

**Fig. 3:**
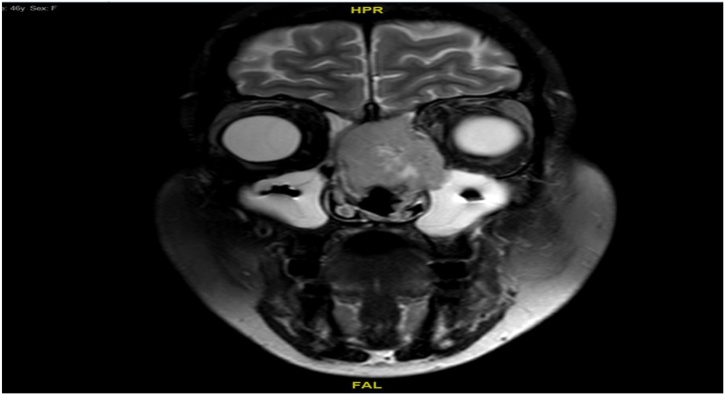
Preoperative MRI T2.

Patient was later admitted for full workup of left nasal high grade spindle cell sarcoma. Sinus magnetic resonance imaging (MRI) revealed left nasal cavity mass expanding to the left frontal recess with extension to the left frontal sinus. The lesion showed involvement of the left lamina papyracea and cribriform plates with small extension to the left intra-orbital compartment. The lesion showed an intermediate to low signal lesion in T2 weighted images with heterogeneous enhancement. It was approximately 3 × 2.7 × 3.5 cm in dimension (Fig. 3). Neck CT showed prominent upper cervical lymph nodes. Chest, abdomen and pelvis CT were clear from any metastasis.

Patient’s clinical, radiological and pathological results were discussed in tumor board. Final decision with medical oncology, radiation oncology, and neurosurgery was to go for surgery followed by adjuvant chemoradiotherapy.

Rheumatology advised to stop Methotrexate 2 weeks prior to surgery.

Patient underwent anterior cranial tumor resection, bicoronal craniotomy and skull base reconstruction, by otolaryngologist and neurosurgeon.

Left external ethmoidectomy, left medial maxillectomy, bilateral sphenoidotomy were performed with ligation of the left anterior ethmoidal artery. lamina papyracea and periorbital fat on the left side were resected. The nasal septum was separated from the face of sphenoid and maxillary crest with anterior preservation of the nasal caudal septum. Bicoronal incision, pericranial flap and frontal craniotomy were performed. Resection of the tumor from the anterior cranial fossa was done; at the area from the right cribriform plate to the left fovea ethmoidalis, anterior to the level of crista galle and posterior to the face of sphenoid. Left temporalis muscle fascia was harvested to reconstruct the left periorbital area.

**Fig. 4:**
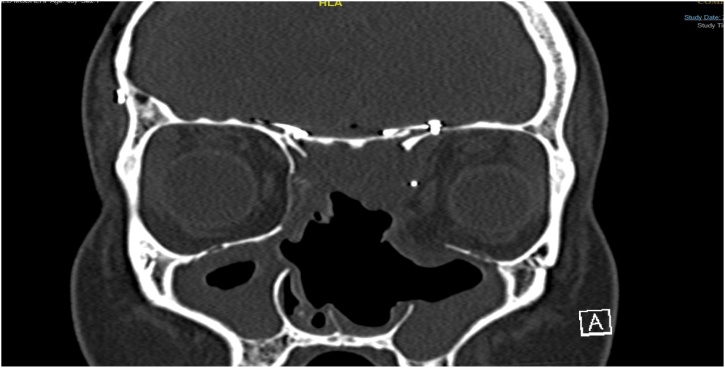
Postoperative CT.

Post operation, patient was kept intubated in the intensive care unit (ICU). CT done day one post operation and revealed no intracranial haemorrhage (Fig. 4). Patient was extubated and taken to a regular ward. Then, patient developed central diabetes insipidus and was treated by one dose of desmopressin. Family noticed behavioral changes in the patient and new brain CT revealed right frontal hemorrhage and edema that improved with time.

Patient was discharged home on second week after surgery with normal facial nerve and vision, healing wound and stable condition. She was given follow up with otorhinolaryngology, neurosurgery, medical and radiation oncology and rheumatology.

Patient completed full course of adjuvant radiotherapy with monthly follow up in otorhinolaryngology clinic for debridement and examination.

Seven months post-operation, findings using 30 degree rigid scope revealed healed nasal cavity mucosa except for a small frontal area with no water discharge.

**Fig. 5:**
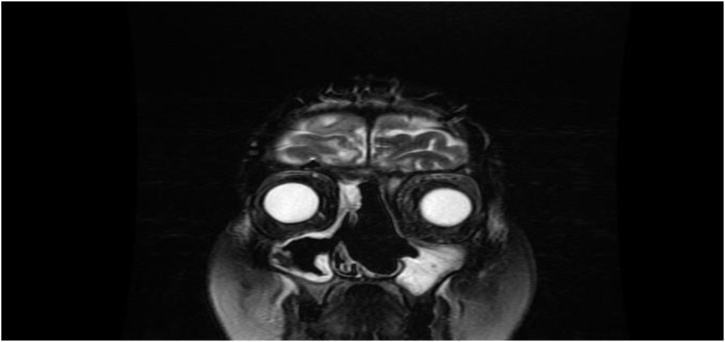
Postoperative and adjuvant radiotherapy, MRI T2.

Patient is following on regular basis with new MRI every 4 months with no evidence of local recurrence (Fig. 5).

## 3 Discussion

Synovial sarcoma is a mesenchymal spindle cell tumor found in soft tissue and unlike its name is not related to synovial membrane. It originates from embryogenic mesoderm [1,5]. It is considered as a rare tumor accounting for up to 1% of all head and neck primary tumors. It accounts 5–10% of all adult sarcoma and around 5–15% of synovial sarcoma can be found in head and neck areas. While in pediatric population, head and neck sarcoma represents around 35% of all sarcomas [[1], [2], [3],^5^]. Harb et al. reviewed 44 cases of synovial sarcomas in the head and neck and revealed that 73% of their patients were male with a median age of 29 year of age, similar findings to the review done by Saito et al. [2,6].

In literature, synovial sarcoma is described as a high grade malignant tumor with the paraspinal neck being the most common location of this neoplasm in the head and neck region. Nevertheless, it could also be found in the nasopharynx, oropharynx and thyroid gland in some cases with rare occasions found in nasal cavity and maxilla similar to our presented case.

Presenting symptoms are mostly determined by location of tumor [[1], [2], [3]]. Reddy et al. described nasal manifestations ranging from nasal obstruction, discharge, foul smell to epistaxis [1].

No specific etiology has been attributed to the development of synovial sarcoma. However, radiation exposure was found to have an obvious association [3].

A spectrum of histopathological variations of synovial sarcomas have been described from monophasic, biphasic to poorly differentiated ones and many others [1,2,6]. Categorizing the lesion based on histopathology may help in immunohistochemistry and molecular targeted therapies [1].

Diagnosis of similar cases is mostly done by radiographic images of paranasal sinus CT. Further diagnostic tools such as MRI and histopathological examination are needed to confirm the diagnosis and rule out further extension.

The differential diagnosis of tumors in this location includes rhabdomyosarcoma, Ewing sarcoma, malignant peripheral nerve sheath tumor and melanoma, which are less likely in the presented case based on the histology and immunoprofile status.

The mainstay treatment of a nasal and other head and neck synovial sarcoma is total excision of the lesion by different modalities with consideration of chemoradiotherapy. Surgical approach depends on tumor location and its extension varying from direct endoscopic resection to craniofacial approaches. Diaz et al’s case of nasal synovial sarcoma was approached by complete endoscopic resection of the mass, ethmoids, medial walls of maxillary sinuses, anterior and medial walls of sphenoid sinuses and orbital extension of the tumor [2,3]. No reported data for most common post-operative complications was found in literature. In our presented case, patient developed central diabetes insipidus which was managed accordingly.

The role of adjuvant chemoradiotherapy varied in literature. Saito et al. reviewed 11 cases of head and neck synovial sarcomas, seven of which had adjuvant radio and/or chemotherapy with no reported therapeutic response [2]. However, Gopalakrishnan et al. reviewed 44 cases of head and neck synovial sarcomas and concluded that chemotherapy has little effect on overall survival as compared to surgical resection [6].

Prognosis of head and neck synovial sarcoma depends on size, location and extension of the tumor. Nevertheless, it was found to have similar rates of all sarcomas with a 5 year-survival rate reaching up to 50% [2,[6], [7], [8]].

## 4 Conclusion

Synovial sarcoma is a rare mesenchymal synovial tumor found in soft tissue. The case presented here highlights an especially rare situation of this tumor involving nasal cavity synovial sarcoma with presenting symptom of recurrent epistaxis. It stresses on the importance of early detection of this tumor for better management. Thus, it is crucial to be aware of the clinical, imaging and histopathological features of this tumor in head and neck for better diagnosis and treatment of the variable craniofacial neoplasm. Furthermore, reporting of this rare tumor should be encouraged for further more accurate epidemiological, clinical, management and prognostic data.

## Declaration of Competing Interest

No conflicts of interest.

## Sources of funding

No source of sponsorship.

## Ethical approval

Research has been approved by the Research Ethical Committee of King Abdullah International Medical Research Center.

## Consent

Consent was taken from the patient for publication purposes.

## Author contribution

Salwa AlRashed ALHumaid: Data collection, data analysis & interpretation and writing the paper.

Mohammad Almahdi: Study concept & design, data collection, data analysis or interpretation, writing the paper.

Maryam Alsafi: Data collection, data analysis & interpretation and writing the paper.

Fahad ALObaid: Data analysis & interpretation and writing the paper.

Alaa Alsalim: Data analysis & interpretation and writing the paper.

## Registration of research studies

Non Applicable.

## Guarantor

Mohammad Almahdi.

Salwa ALRashed ALHumaid.

## Provenance and peer review

Not commissioned, externally peer-reviewed.
